# Simulations of the Behaviour of Steel Ferromagnetic Fibres Commonly Used in Concrete in a Magnetic Field

**DOI:** 10.3390/ma15010128

**Published:** 2021-12-24

**Authors:** Kateřina Nováková, Kristýna Carrera, Petr Konrád, Karel Künzel, Václav Papež, Radoslav Sovják

**Affiliations:** 1 Department of Electrotechnology, Faculty of Electrical Engineering, Czech Technical University in Prague, Technická 2, 166 27 Prague, Czech Republic; novakk25@fel.cvut.cz (K.N.); kuenzel@fel.cvut.cz (K.K.); papez@fel.cvut.cz (V.P.); 2 Experimental Centre, Faculty of Civil Engineering, Czech Technical University in Prague, Thákurova 7, 166 29 Prague, Czech Republic; kristyna.carrera@fsv.cvut.cz (K.C.); sovjak@fsv.cvut.cz (R.S.)

**Keywords:** concrete, steel, fibre, orientation, magnetic, simulation, interaction

## Abstract

The efficiency of fibre reinforcement in concrete can be drastically increased by orienting the fibres using a magnetic field. This orientation occurs immediately after pouring fresh concrete when the fibres can still move. The technique is most relevant for manufacturing prefabricated elements such as beams or columns. However, the parameters of such a field are not immediately apparent, as they depend on the specific fibre reaction to the magnetic field. In this study, a numerical model was created in ANSYS Maxwell to examine the mechanical torque acting on fibres placed in a magnetic field with varying parameters. The model consists of a single fibre placed between two Helmholtz coils. The simulations were verified with an experimental setup as well as theoretical relationships. Ten different fibre types, both straight and hook-ended, were examined. The developed model can be successfully used to study the behaviour of fibres in a magnetic field. The fibre size plays the most important role together with the magnetic saturation of the fibre material. Multiple fibres show significant interactions.

## 1. Introduction

Fibre-reinforced concrete is one of the modern composite materials that benefits concrete structures [1,2]. The main advantages of using fibres in concrete are resistance to extreme loadings and increased tensile strength [3,4,5,6]. Another point is the possibility of orienting fibres and thus obtaining anisotropic properties when needed, particularly a targeted increase in tensile strength and the capacity to dissipate mechanical energy in the desired direction [7,8,9,10,11]. In 3D printing applications, fibre orientation also plays a key role [12,13,14,15]. Many methods can be used to achieve fibre orientation, notably the casting technique of fresh concrete [16,17,18]. In the case of steel fibres, magnetic field orientation can be used. Such orientation has been explored in several studies [19,20,21,22,23]. These studies described the basic principles and theoretical application, but several aspects of the fibre orientation process are still being researched. The significance of the presented research is in a more detailed description of the specific behaviour of various types of steel fibres in the magnetic field, so that the results may serve as a basis for further development of the final devices and techniques for magnetic orientation.

The force, or torque applied to the fibre, is a critical parameter for successful fibre orientation using a magnetic field. The magnetic field effects must be strong enough to enable the fibres to overcome the resistance of the fresh mixture and orient themselves in the desired direction. The force with which the fibre can rotate in fresh concrete depends not only on the strength of the magnetic field but also on its material (i.e., whether it is a magnetically hard or soft material), whether the fibre has been pre-magnetised or not, and its dimensions and geometry [24]. The fibres commonly used in concrete vary in a wide range of sizes and shapes. In addition to straight fibres, fibres with hooked-ends are also used, which improve the fibre anchoring for higher crack-bridging efficiency. In this study, we aimed to examine the behaviour of different types of fibres in a magnetic field through numerical simulations. The results of these simulations will help to more efficiently prepare experimental work and better set the experimental conditions. Finally, appropriate simulations will allow the investigation of fibre interactions, both desirable and undesirable. Concerning the analysis of the electromagnetic properties of the system, ANSYS 2021 R1 [25] was used, namely its component ANSYS Maxwell, to create a finite element method (FEM) model [26,27,28]. This software is suited for studying electromechanic systems in the range of low-frequency electromagnetic fields. The benefit of simulations is the possibility of choosing different layouts and verifying the functionality before the actual implementation of the device. In this paper, the geometrical arrangement corresponds to an experimental measurement setup where the motion of the fibres in a magnetic field was monitored.

## 2. Materials and Methods

**Figure 1:**
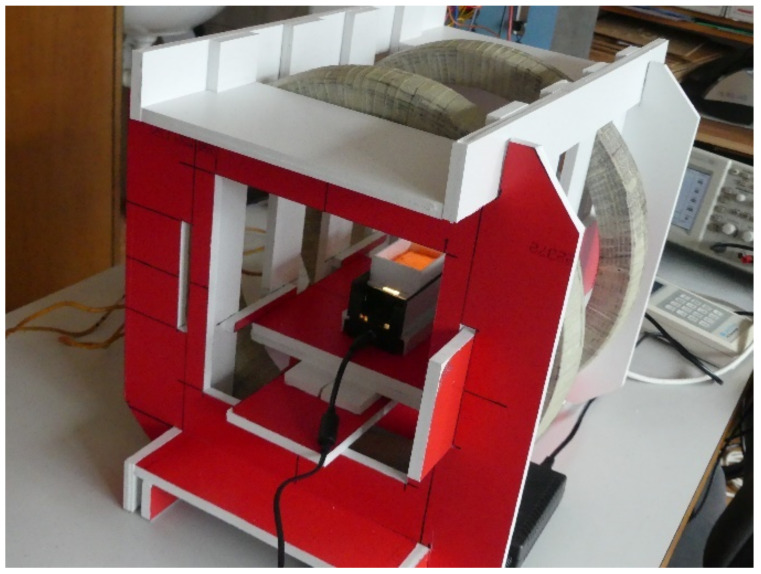
Experimental measuring setup.

Figure 1 shows the experimental setup. It consisted of two identical coils with an outer diameter of 390 mm with a rectangular cross-section of 40 mm × 45 mm and windings wound with 260 turns arranged as Helmholtz coils [29,30] to achieve a uniform magnetic field in the space between them. Various fibres embedded in an ultrasound gel were inserted into this space, and their motion was monitored. The ultrasound gel was chosen as a cement mixture replacement [31] to allow a visual analysis of the position of the fibres. All other materials of the measurement assembly were chosen to be nonmagnetic (mostly foamed PVC) to not affect the magnetic field. This coil configuration was chosen to create a homogeneous magnetic field of sufficient intensity around the fibre in order to study the field’s effects. The specific parameters of the coils were chosen based on trials and measurements of the necessary magnetic field’s strength and shape.

**Figure 2:**
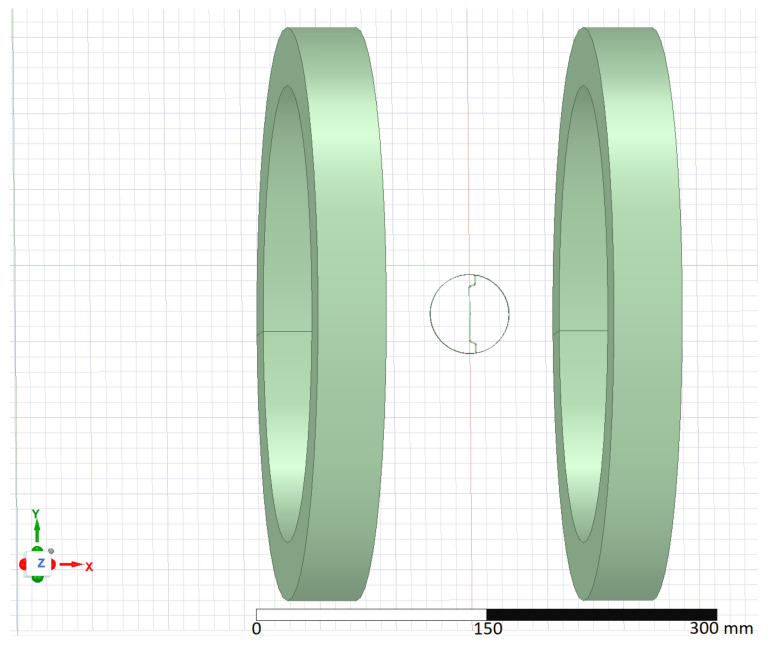
The simulation setup in ANSYS.

The geometric setup simulated in ANSYS was identical to the experimental workplace, as shown in Figure 2. The main working parts were two cylindrical coils and the affected fibre (or several fibres).

**Figure 3:**
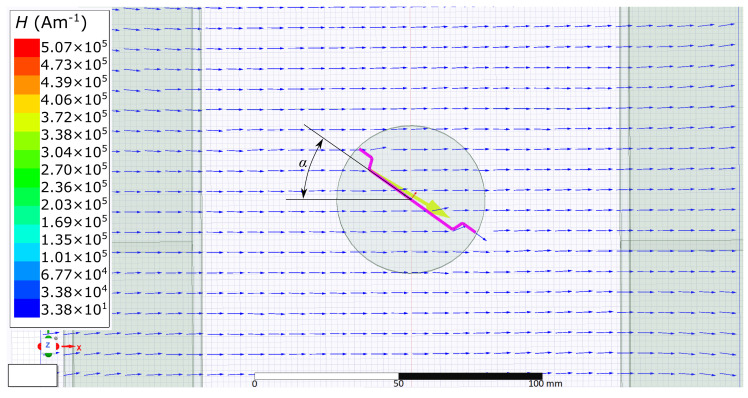
Magnetic field distribution around the fibre and fibre angle definition.

**Figure 4:**
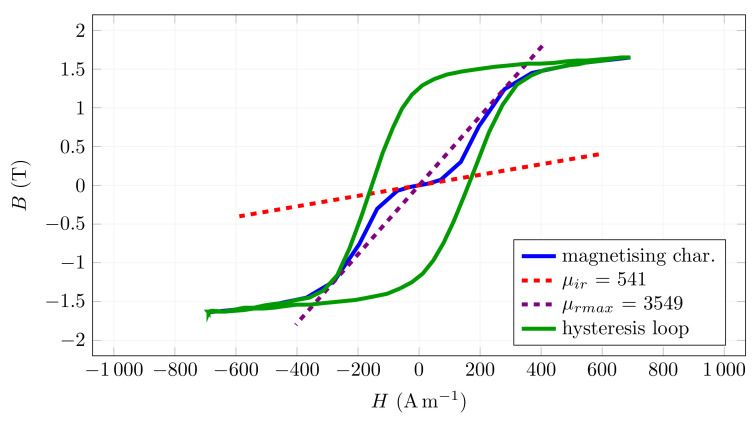
An example of the magnetic characteristics and hysteresis loop of Dramix 3D 55/30 B fibre, where $\mu_{ir}$ is the initial relative permeability and $\mu_{rmax}$ is the maximal relative permeability.

During the simulation, the field distribution around the fibre and in the fibre was observed, and the dependence of the torque on the angle of the fibre rotation concerning the magnetic field axis was investigated (Figure 3). Verification of the simulated results is described in the following sections. For the purpose of the simulations, the measured parameters of the fibres, i.e., their magnetisation characteristics and hysteresis loop, were used in the simulations from a previous study [24]. An example of these parameters is shown in Figure 4.

The magnitude of the magnetic induction *B* (T) in the axis between the coils was also verified with the calculation of the magnetic induction obtained from the well-known derivation for Helmholtz coils:

(1) $$B={\left(\frac{4}{5}\right)}^{\left(\frac{3}{2}\right)}\frac{\mu_{0}NI}{R}$$

where $\mu_{0}$ (Hm${}^{-1}$) is the permeability of free space, *N* is the number of turns, *I* (A) is the electrical current, and *R* (m) is the radius of the coil. As such, the functionality and correctness of the simulations were verified.

## 3. Results and Discussion

### 3.1. Simulation of Weak Magnetic Field

Most of the steel fibres used in concrete have the properties of soft magnetic steel. The hysteresis loop of such materials is relatively wide, and the initial permeability is relatively low. The measurements revealed that the initial relative permeability of the material $\mu_{ir}$ is in the range of 400 to 600 [24] for the tested fibres. In the region of initial magnetisation, the fibre shows practically linear dependence between magnetic induction and magnetic field strength *H* (Am${}^{-1}$), i.e.,

(2) $$B=\mu_{i}H$$

where the permeability of the material is practically constant, as shown in Figure 4.

For the mechanical moment $\vec{M}$ (Nm) acting on a fibre, we can use the magnetic moment $\vec{m}$ (Am${}^{2}$) relationship [32]

(3) $$\vec{M}=\vec{m}\times \vec{B}$$

to simply derive that it will be dependent on the square of the intensity of the magnetic field and the sine of double the fibre angle α (rad). For small values of the magnetic field intensity, we can verify the functionality of the model, where the results should approach the limit calculated using:

(4) $$\left|M\right|=\frac{1}{2}Sl\frac{\mu_{ir}}{\mu_{0}}{\left|B\right|}^{2}\sin2\alpha$$

where *S* (m${}^{2}$) is the fibre’s cross-section and *l* (m) is the fibre’s length. The correctness of the model and the correct specification of the magnetic material parameters can be checked with these equations.

**Figure 5:**
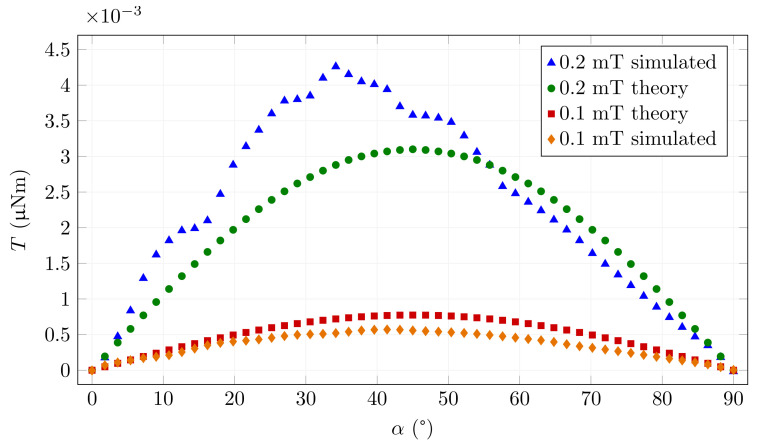
An example of the dependence of the mechanical torque acting on a Weidacon FM fibre on the angle of rotation when the magnetic induction in the air around the fibre is *B*${}_{0}$ = 0.1 mT and *B*${}_{0}$ = 0.2 mT.

Figure 5 shows the mechanical torque applied to Weidacon FM fibres at the magnetic induction *B*${}_{0}$ = 0.1 mT as a function of the angle. For comparison, the theoretical limiting function according to Equation (4) is also plotted for identical fibre dimensions for constant $\mu_{ir}$ = 528.

The plot shows the function for very low saturations on the linear part of the magnetic characteristic (for 0.1 mT). The graph for 0.2 mT is also shown for comparison. In this case, the magnetisation characteristic bends, and the relative permittivity increases significantly. The magnetic field in the fibre is thus higher. The simulated torque function then deviates correctly from the torque calculated with Equation (4) for a small initial permeability. The resulting curve obtained by simulation respects this process, and the torque reaches higher values, as expected.

For larger rotation angles, the magnetic induction in the fibre is lower and enters a mostly linear region. The above-mentioned action is conditioned by the fact that we move along the magnetisation curve from lower to higher values, i.e., we are assuming an unmagnetised fibre that we rotate from perpendicular to parallel position relative to the direction of the magnetic field or we are examining individual points on the curve from an unmagnetised state. If this condition is not met, then the hysteresis loop applies, which is different from the magnetisation curve.

### 3.2. Simulation of Magnetically Saturated Fibres

The second limiting case occurs if the magnetic field is strong enough so that the magnetic saturation of the fibre material occurs. The magnetic induction in the fibre is then practically constant and can be considered as *B*${}_{f}$ = *B*${}_{fmax}$ as if it were a permanent magnet oriented in the fibre axis. The theoretical mechanical moment acting on the fibre can then be derived as a magnetic dipole moment:

(5) $$\left|M\right|=\frac{Sl}{\mu_{0}}B_{fmax}Bsin\alpha$$

where *B*${}_{fmax}$ (T) is the magnetic induction of a saturated fibre and *B* (T) is the magnetic induction between the coils in air around the fibre.

**Figure 6:**
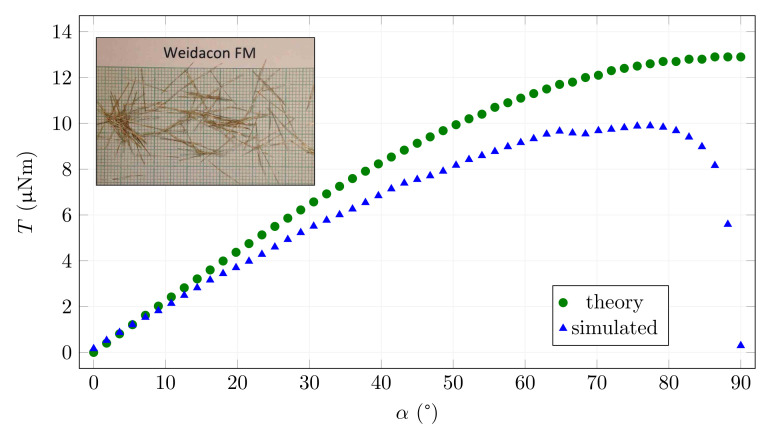
An example of the dependence of the mechanical torque acting on a saturated Weidacon FM fibre (*B*${}_{fmax}$ = 1.76 T) on the angle of rotation when the magnetic induction in the air around the fibre is *B*${}_{0}$ = 25 mT.

This theoretical function represents a sinusoidal dependence on the rotation angle. In practice, the measured data and the simulated data should deviate from the theoretical characteristic, especially in the region where the fibre is oriented perpendicular to the magnetic flux and in positions close to it. For other angles, we expect values lower but close to the theoretical ones. An example of the resulting dependence of torque on the rotation angle is shown in Figure 6 together with a comparison to the theoretical limiting values of Equation (5). The pictures embedded in this and later figures show examples of the fibres. The results in the graph show the correctness of the assumptions and the functionality of the model.

This example is, for practical reasons, more interesting because the ferromagnetic material of the fibre presents a significantly lower magnetic resistivity for the magnetic flux, so the magnetic field strength in the fibre is considerably higher than in the surrounding air. Moreover, the values of magnetic induction required to induce sufficient torque to orient the fibre in the cement mixture occur in this region. It can be seen from the graph that the simulation results are close to the idealised function. For low rotation angle values, the fibre is strongly oversaturated and the two curves practically overlap. With increasing fibre rotation, the chart diverges as the fibre’s longitudinal magnetic induction decreases. For angles above 80${}^{\circ }$, the above assumption of magnetic saturation of the fibre in its longitudinal axis is no longer valid. The results differ more, as expected, when the mechanical torque drops to zero for the perpendicular direction. The idealised function assumes the same behaviour as a permanent magnet, i.e., it reaches a maximum in the perpendicular direction.

### 3.3. The Effect of Computational Mesh Size

**Figure 7:**
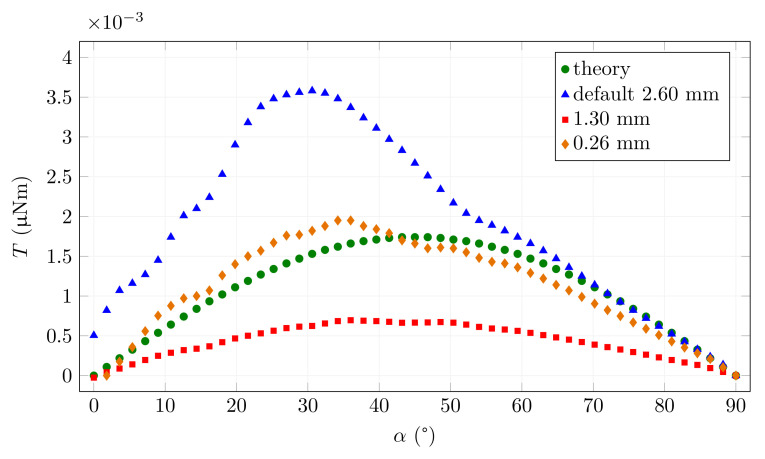
The effect of computation maximum mesh size.

**Figure 8:**
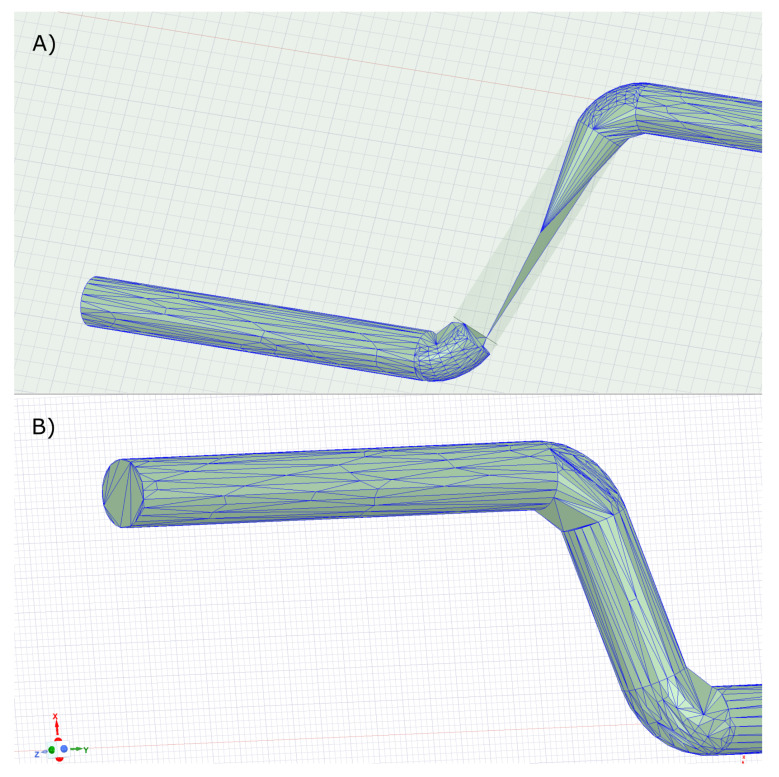
Examples of fibre meshes when (**A**) the default maximum mesh size was chosen and (**B**) when the maximum mesh size was limited to 0.26 mm.

Another possible negative effect on the simulation may be the choice of mesh size for the calculation. This is mainly due to the large dimensions of the whole device relative to the small fibre. A coarse mesh in fibre may distort the simulation results. The effect on results is illustrated in Figure 7. If the default option is chosen, the maximum size of the mesh element is larger than the fibre diameter (Figure 8A). This will cause the magnetic field as well as the torque in the fibre to be miscalculated. Only when the maximum element size is chosen appropriately relative to the thickness of the fibre do we obtain reasonable results, which then do not change with further refinement (less than 0.26 mm, Figure 8B) of the calculation mesh. Too fine a mesh size leads to an unnecessarily increased computation time. For comparison, the theoretical function corresponding to the simulated low saturation is also shown in the graph. For all simulations presented in the paper, the 0.26 mm maximum mesh size was used.

### 3.4. The Results of Commonly Used Fibres

After verifying the functionality of the model, the torque acting on commonly used fibres was investigated. An overview of the simulated fibres is provided in Table 1.

**Figure 9:**
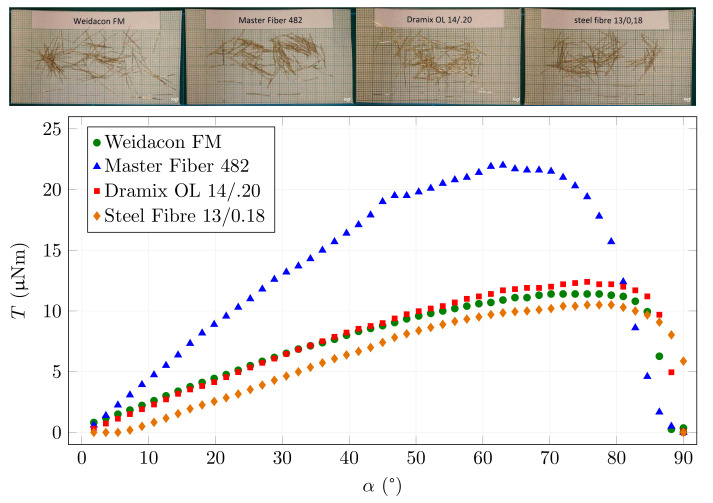
Torque and fibre angle relationship for different types of straight fibres.

The following results show the dependence of the mechanical torque acting on a single fibre placed in a magnetic field of a given intensity ($B_{0}$ = 25 mT) on the angle of rotation. The first graph in Figure 9 shows four types of straight fibres. At first glance, it can be seen that the results are consistent with the derived equation because the achieved torque on the fibre depends mainly on the product of the cross-section and the fibre length. The three close curves belong to fibres with similar dimensions. The differences in the magnetisation curves are not so significant as to outweigh the effect of the fibre dimensions. The highest curve is obtained by Master Fibre 482, which has approximately twice the cross-section of the other fibres. In addition to almost doubling the torque, these fibres experience a decrease in magnetic induction in the fibre at a smaller rotation angle than the other three fibre types. This result corresponds to the different cross-sections of the fibre at the same length.

**Figure 10:**
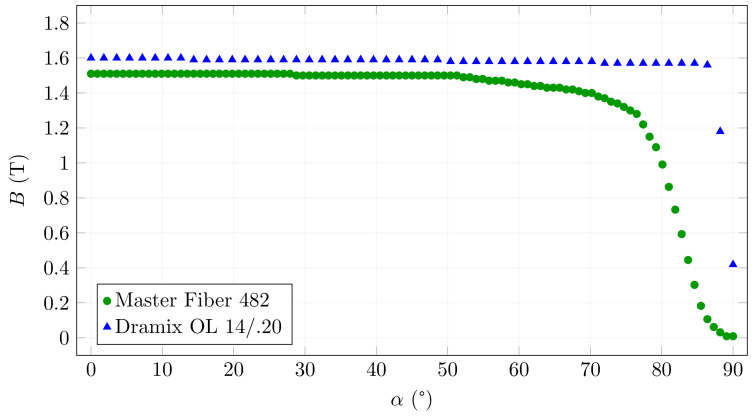
Magnetic induction in the centre of the fibre depending on the angle of rotation for *B*${}_{0}$ = 25 mT.

The different behaviours of the Master Fibre 482 can also be illustrated by the fibre saturation differing with increasing rotation angle. Figure 10 shows the magnetic induction in the centre of two fibre examples. It can be seen that Master Fibre 482 already exhibits a decrease in magnetic induction at around 50${}^{\circ }$, compared to the constant values up to around 85${}^{\circ }$ for Dramix OL 14/0.20.

**Figure 11:**
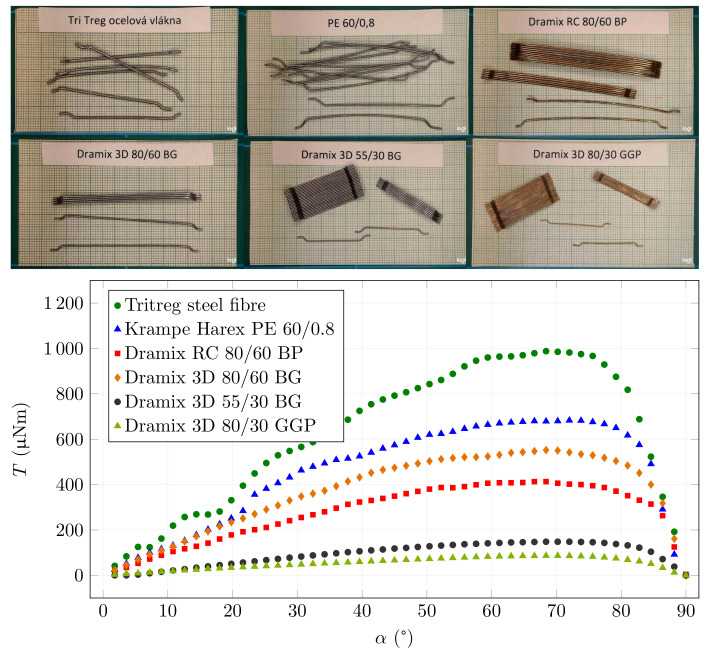
Torque and fibre angle relationship for different types of hook-end fibres.

Figure 11 shows the results for fibres with larger sizes and hooked-ends. These fibres are sometimes supplied in bundles connected by water-soluble glue, as shown in the embedded pictures in Figure 11. These fibres are designed to withstand higher pull-out forces due to their stronger anchoring in the cement matrix. These results were also obtained for $B_{0}$ = 25 mT. The curves shapes are similar for all types. The only significant difference is the scale. The theoretical relationship from Equation (5) depends on the product of cross-section area and length, i.e., the volume of the fibre. If we compare the values of volume from Table 1 and the scales of the curves, we can clearly see that the results are in line with this theoretical derivation.

### 3.5. Examples of Fibre Interaction

**Figure 12:**
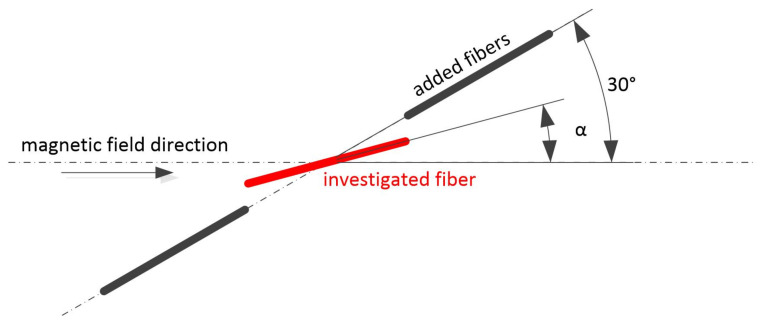
The arrangement of fibres used to investigate fibre interactions.

Once the simulations with one fibre are verified, the model can be extended to simulate more fibres. An example of this can be the chaining behaviour where multiple fibres may randomly come together to form a connection that affects the original magnetic field. Thus, the resulting orientation may not achieve the desired parameters. An example of such off-axis chaining is shown in Figure 12. The positions of two fibres are chosen to be fixed at an angle of 30${}^{\circ }$. They form a chain with the fibre under examination, i.e., the centre of the fibre under investigation is placed on a common axis in the centre between the two fibres. When the orientation is identical, there is a small gap of 0.2 mm between the fibres.

**Figure 13:**
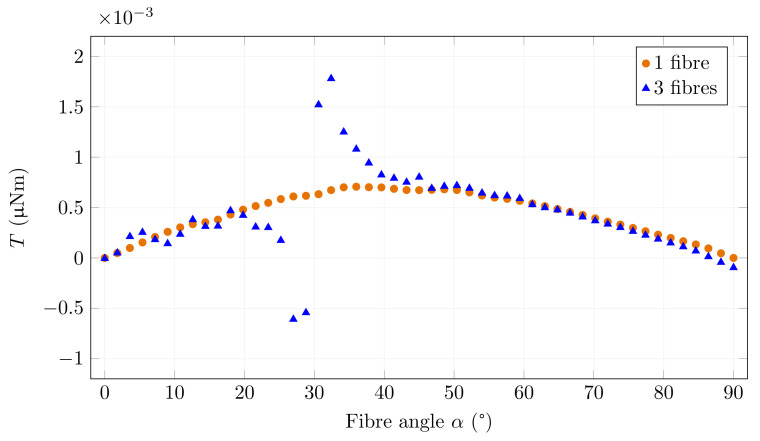
Torque and fibre angle relationship for one and three fibres for *B*${}_{0}$ = 0.1 mT.

**Figure 14:**
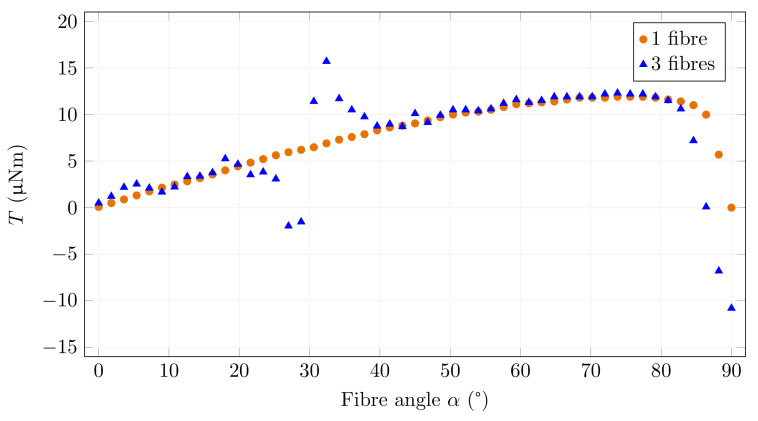
Torque and fibre angle relationship for one and three fibres for *B*${}_{0}$ = 25 mT.

The results are shown in Figure 13 and Figure 14. These two graphs differ in the magnetic induction generated by the coils. Figure 13 shows the simulation for $B_{0}$ = 0.1 mT when the fibres are still in the unsaturated part of the magnetisation characteristic. It is clear from the chart that the magnetic field around the investigated fibre is deformed for all rotation angles. A significant difference is observed around 30${}^{\circ }$ when the field is oriented and amplified by adding fibres that act as pole extensions. The torque acting on the fibre tries to turn it on the same axis as the added fibres. Due to the reinforcement of the field by the inserted fibres, this torque is significantly higher than the original torque acting on the single fibre.

The fibres become saturated when the strength of the magnetic field increases (Figure 14). In this case, the difference between the torque acting on a single fibre and the torque of the field deformed by the added fibres is smaller. The reason for this phenomenon is that the torque depends on the magnetic induction in the fibre. The magnetic induction increases in the nonsaturated state. However, it is practically constant in the saturated state except for angles close to 90${}^{\circ }$. Nevertheless, the tendency to align with the other fibres is present.

## 4. Conclusions

In this paper, we presented the numerical simulations of the behaviour of steel fibre in a magnetic field. The results showed that the FEM models are applicable for this kind of interaction. The most important feature of these simulations is the ease of parameter changes, so larger coils can be designed with the knowledge of the strengths of the magnetic fields necessary for specific fibre types and the desired effect. However, the models must be constructed with the knowledge of the magnetic parameters of the fibres and sufficiently fine computational mesh must be used; otherwise, the results can be significantly inaccurate, as presented in the Results and Discussion. The most important parameter of the behaviour is the fibre geometry; however, the magnetisation characteristics should also be taken into account. The correct behaviour of the model can be verified using theoretical assumptions.

Single fibre models are necessary before conducting multiple fibre simulations. From the presented results, it is clear that fibre interactions play a key role in the overall response to the magnetic field. For the investigation of more complicated spatial combinations of fibres, the model was developed from the beginning as three-dimensional, although in the cases presented here, this was not necessary. This work may significantly influence the progress in the relatively new technology of reinforced concrete with magnetic-field-oriented steel fibres. Further work on the model will be aimed toward simulating the rheological properties of the cement mixture.

## Figures and Tables

**Table 1:** Overview of the used fibres and their selected parameters. $H_{c}$ is the magnetic coercivity and $B_{800}$ is the magnetic flux density inside the fibre for field strength of 800 Am${}^{-1}$. Commercial names written in the Fibre Type column, manufacturers information in the table’s footnotes.

Fibre Type	Length	Diameter	Volume	$\mu_{\mathrm{ir}}$	$H_{c}$	$B_{800}$
	(mm)	(mm)	(mm${}^{3}$)	-	(Am${}^{-1}$)	(T)
Master Fiber 482 ^1^	13	0.32	1.05	388	150	1.60
Dramix OL 14/0.20 ^2^	13	0.22	0.49	512	164	1.59
Dramix 3D 55/30 BG ^2^	30	0.56	7.39	541	161	1.70
Dramix 3D 80/30 GGP ^2^	30	0.38	3.40	488	154	1.79
Dramix 3D 80/60 BG ^2^	60	0.75	26.51	547	192	1.77
Dramix RC 80/60 BP ^2^	60	0.72	24.43	428	182	1.70
Steel Fibre 13/0.18 ^3^	13	0.185	0.35	469	146	1.79
Tritreg Steel Fibers ^4^	50	1.05	43.30	605	203	1.83
Krampe Harex PE 60/0.8 ^5^	60	0.784	28.96	555	199	1.75
Weidacon FM ^3^	13	0.19	0.37	528	188	1.76

^1^ Master Builders Solutions CZ s.r.o., K Májovu 1244, 537 01 Chrudim, Czech Republic. ^2^ Bekaert Petrovice s.r.o., Petrovice 595, 735 72 Petrovice u Karviné, Czech Republic. ^3^ StraTec Strahl- und Fasertechnik GmbH, An der Schleuse 3, 58675 Hemer, Germany. ^4^ Tritreg—Tˇrinec, s.r.o., Frýdecká 390, 739 61 Tˇrinec, Czech Republic. ^5^ KrampeHarex CZ s.r.o., Osvobození 234, 664 81 Ostrovaˇcice, Czech Republic.

## Data Availability

Data are contained within the article.
